# Dr. Amick and the Lancet-Clinic

**Published:** 1892-04

**Authors:** 


It is but fair to the *Lancet-Clinic* to say that from reliable information received in correspondence with a prominent medical gentleman of Cincinnati, in no way connected with that journal, the *Lancet-Clinic* was not only imposed upon by Dr. Amick in procuring the publication of that long article on his so-called "Chemical Cure for Consumption" with the long list of almanac-like certificates, but he abused the confidence of the editor.

It seems Dr. Amick was an occasional contributor to the *Lancet-Clinic*, and his articles heretofore having been correct and acceptable, when he handed in the rig-a-marole the editor, without examining it, sent it to the printers. Dr. Amick's office being very near the printing office, the proof was sent to him for correction and back to the "make-up." Hence, it got into the *Lancet-Clinic* without a knowledge on the part of the editor of its real nature. It is customary with some journals to have contributors read proof of their articles; but the editor should read it also.

That Dr. Richardson did not investigate the matter before publication only goes to show his unbounded confidence in Dr. Amick; and shows, too, that Dr. Amick took advantage of that confidence to inflict this injury upon the journal. It only adds to his disgrace. He has virtually sold his birthright for a mess of pottage. He will be invited to resign the chair of Ophthalmology in the Cincinnati College if he does not hasten to forestall such action by resigning voluntarily. The profession of Cincinnati condemn the publication very generally; and those who hastened to send in vouchers, and certificates from the brick layer who was "delighted," and said the medicine "went right to the spot;" and from the fond parent whose "Willie liked the Chemical Cure better than anything," candy not excepted, are now endeavoring, we learn, to explain how they came to do it.

Prof. Amick, in the meantime, will be relegated to the ranks of the great army of quacks—his proper level, where he will join H. H. Kane and other kindred spirits.

Alas! what is a professor profited if he gain a whole bushel of certificates, and lose the confidence and respect of all reputable medical men?

